# Fibroblast-Derived Extracellular Vesicles Induce Colorectal Cancer Progression by Transmitting Amphiregulin

**DOI:** 10.3389/fcell.2020.00558

**Published:** 2020-07-07

**Authors:** Ádám Oszvald, Zsuzsanna Szvicsek, Márton Pápai, Andrea Kelemen, Zoltán Varga, Tamás Tölgyes, Kristóf Dede, Attila Bursics, Edit I. Buzás, Zoltán Wiener

**Affiliations:** ^1^Department of Genetics, Cell and Immunobiology, Semmelweis University, Budapest, Hungary; ^2^Research Centre for Natural Sciences, Budapest, Hungary; ^3^Uzsoki Hospital, Budapest, Hungary; ^4^MTA-SE Immune-Proteogenomics Extracellular Vesicle Research Group, Semmelweis University, Budapest, Hungary; ^5^HCEMM-SE Extracellular Vesicle Research Group, Budapest, Hungary

**Keywords:** organoid, fibroblast, amphiregulin, exosomes, colorectal cancer, TGFβ

## Abstract

Extracellular vesicles (EV), structures surrounded by a biological membrane, transport biologically active molecules, and represent a recently identified way of intercellular communication. Colorectal cancer (CRC), one of the most common cancer types in the Western countries, is composed of both tumor and stromal cells and the amount of stromal fibroblasts negatively correlates with patient survival. Here we show that normal colon fibroblasts (NCF) release EVs with a characteristic miRNA cargo profile when stimulated with TGFβ, one of the most important activating factors of fibroblasts, without a significant increase in the amount of secreted EVs. Importantly, fibroblast-derived EVs induce cell proliferation in epidermal growth factor (EGF)-dependent patient-derived organoids, one of the best current systems to model the intra-tumoral heterogeneity of human cancers. In contrast, fibroblast-derived EVs have no effect in 3D models where EGF is dispensible. This EV-induced cell proliferation did not depend on whether NCFs or cancer-associated fibroblasts were studied or on the pre-activation by TGFβ, suggesting that TGFβ-induced sorting of specific miRNAs into EVs does not play a major role in enhancing CRC proliferation. Mechanistically, we provide evidence that amphiregulin, transported by EVs, is a major factor in inducing CRC cell proliferation. We found that neutralization of EV-bound amphiregulin blocked the effects of the fibroblast-derived EVs. Collectively, our data suggest a novel mechanism for fibroblast-induced CRC cell proliferation, coupled to EV-associated amphiregulin.

## Introduction

Extracellular vesicles (EV) are membrane-surrounded structures released by virtually all cell types. They transport biologically active molecules (such as nucleic acids, lipids and proteins) from the releasing to the target cells, thus, participating in the recently identified way of intercellular communication (Tkach et al., 2018). Since EVs transport their cargo in a protected way in the tissues and body fluids, furthermore, molecules specific for the releasing tumor cells are represented in them at a high concentration, they provide a promising tool for early cancer diagnostics. EVs form a heterogeneous group of vesicles according to both their size and cellular origin (Thery et al., 2018). Small EVs (sEVs, 35–150 nm), e.g., exosomes are released from the multivesicular bodies of the endosomal-lysosomal system, while medium sized EVs (mEVs, 100–1,000 nm) are commonly shed from the plasma membrane by a budding mechanism (Thery et al., 2018). There are also large EVs (e.g., apoptotic bodies, >1,000 nm) that carry not only molecules but organelles as well (Mathieu et al., 2019). Despite the emerging evidence that EVs contribute to tumorigenesis and play a critical role in the communication among stromal and cancer cells (Maia et al., 2018), changes in their release intensity, cargo in different fibroblast populations, and mechanism of their action on colorectal cancer (CRC) cells is by far not yet understood.

Colorectal cancer is among the most frequent cancer types in the developed world. In more than 80% of CRC patients, mutation in the *APC* gene is an initializing genetic change, leading to the continuous and ligand-independent activation of the Wnt pathway (Kinzler and Vogelstein, 1996). In addition, the oncogenic activation of KRAS leads to the independency of the adenoma cells from external epidermal growth factor (EGF) activity. This adenoma stage can then progress to carcinomas with the accumulation of other genetic changes, such as inactivation of *TP53* and the TGFβ signaling pathway. The recently developed organoid systems represent relevant *in vitro* methods to study human cancers (Bleijs et al., 2019). Importantly, patient-derived cancer organoids maintain the cellular heterogeneity of the original tissue when cultured under well-defined conditions and they provide a valuable tool to monitor tumor progression in human samples as well (Drost et al., 2015; Matano et al., 2015; Bolck et al., 2019).

The accumulation of cancer-associated fibroblasts (CAF), an important and abundant cell type in the stroma, results in a worse patient survival in CRC (Calon et al., 2015). CAFs are often identified by the expression of α-smooth muscle actin (αSMA) or fibroblast−activating protein (FAP). Importantly, TGFβ plays a central role in the activation of fibroblasts in CRC and it induces a specific gene expression program, including the induction of HBEGF, IL-6, and IL-11 expression. IL-11 initializes CRC invasion and metastasis via activating the STAT signaling pathway (Calon et al., 2012). The peri-tumoral fibroblasts (PTF), isolated from the normal colon near to the tumor tissue, are often used as the unactivated control cells for CAFs (Herrera et al., 2018). However, a recent publication comparing the expression profiles of PTFs and CAFs found a low level of difference between the corresponding pairs. In this study αSMA, generally considered as a marker of the activated fibroblasts, was present in PTFs as well (Berdiel-Acer et al., 2014).

Interestingly, CAFs critically contribute to the cellular heterogeneity of CRC and to the acquision of the aggressive cancer stem cell phenotype (Vermeulen et al., 2010; Essex et al., 2019). In addition, we found that intestinal fibroblast-derived EVs carry amphiregulin (AREG), a member of the EGF ligand family, and EVs have a central role in shaping the intestinal stem cell niche (Oszvald et al., 2020). However, the role of EVs as conveyors of messages in the stroma-CRC cell communication is not well understood.

## Materials and Methods

### Cell Culture

SW1222 CRC cells were obtained from ECACC (European Collection of Authenticated Cell Cultures) and they were cultured in DMEM containing 4,500 g/L glucose (Gibco), 10% FBS (Biosera), glutamine (Sigma), and 1× penicillin/streptomycin (Gibco). Human colon fibroblasts (American Tissue Culture Collection, ATCC-1459) (NCF) were cultured in fibroblast medium containing DMEM high glucose (containing 4,500 g/L glucose, Gibco), 10% FBS, glutamine and Penicillin/Streptomycin. Cells were washed with phosphate buffered saline (PBS) three times and cultured in serum-free medium or in CRC medium for 2 days before collecting/measuring EVs. CRC medium contained advanced DMEM/F12, 1× N2 and 1× B27 supplement (Gibco), 1 mM N-Acetyl-Cysteine, 10 mM HEPES (Sigma), penicillin/streptomycin, antibiotic/antimycotic mix (Gibco), and glutamine. Cell cultures were tested for mycoplasma with Hoechst staining and they were negative in our studies. We only used cells with <p9 passage numbers after obtaining from ATCC or ECACC. Cell number was determined in a Burker chamber. In some experiments cells were activated with 10 ng/mL TGFβ1 (Peprotech) for 4 days (Calon et al., 2012).

### Isolation of Human Colon Fibroblasts (PTF and CAF)

The Ethics Committee of the Medical Research Council of Hungary (ETT-TUKEB, No. 51323-4/2015/EKU) approved all experiments with human samples and informed consent was obtained from the patients. Samples were collected at the Department of Oncosurgery, Uzsoki Hospital, Budapest, Hungary. Patient data are shown in Supplementary Tables S1, S2. Tumor and normal colon tissue dissected at a distance >5 cm from the tumor were isolated and cut into small pieces (<0.5 cm) in PBS. After extensive washing with PBS three times, tissue pieces were incubated in a digestive mix [DMEM high glucose with 20% FBS, 75 U/mL collagenase type IX (Sigma), and 125 μg/mL dispase type II (Invitrogen, Carlsbad, CA, United States)] for 2 h at 37°C with extensive shaking. After removal of tissue pieces, single cells were then centrifuged at 300 *g* for 5 min, washed twice in PBS and cultured in tissue culture plates (Eppendorf) in fibroblast medium containing 15% FBS.

### Wound Healing Assay

Normal colon fibroblasts, PTFs or CRC-Fs were cultured in the wells of 24-well plates until confluence. A scratch was created with a 200 μL pipette tip. Cells were washed with PBS to remove cell debris and they were cultured in 500 μL medium (DMEM high glucose with 2.5% EV-free FBS, antibiotic/antimycotic mix, and glutamine). In some experiments, cells were pre-treated with TGFβ (10 ng/mL, 4 days). Images were taken at 0, 16, and 24 h (Nikon Diaphot microscope) and the areas without cells were evaluated by the ImageJ software. Data are presented as relative percentage compared to the wound size at the starting time point. For determining the effect of TGFβ, this relative percentage was compared to the control value.

### Detection of SA-β-Galactosidase Activity

Cells were cultured in chamber slides (Falcon), they were fixed with 4% paraformaldehyde (PFA) for 5 min and then washed with water. Samples were incubated in staining solution to detect the enzymatic activity according to (Debacq-Chainiaux et al., 2009) at 37°C in the dark without CO_2_. The reaction was stopped by changing the solution to water after 8 h.

### Human Colon Organoid Cultures

Tumor samples from CRC patients were processed according to previously published protocols (Sato et al., 2011; van de Wetering et al., 2015). The small cell clusters were isolated by centrifugation at 300 *g* for 2 min and they were embedded into Matrigel (Corning). In addition, we used patient-derived organoid lines previously established in our laboratory (Szvicsek et al., 2019). Organoids were cultured in CRC medium supplemented with 500 nM A83-01 (Sigma), 10 μM SB202190-Monohydrochloride (Sigma), and 50 ng/mL EGF. Furthermore, the Rho kinase inhibitor Y27632 (Sigma) was added for 2 days after splitting. Organoids were removed from Matrigel in every 5–6 days mechanically, they were centrifuged at 300 *g* for 5 min and then disrupted by pipetting and digesting with TrypLE (Thermo Fisher, Waltham, MA, United States) for 3–5 min. Cell clusters were then washed and embedded into Matrigel again. Clinical data of the CRC patients and characterization of CRC organoids #1–3 have been published (Szvicsek et al., 2019; Supplementary Table S1).

### EV Isolation

Fibroblasts were cultured in CRC medium for 4 days with or without 10 ng/mL TGFβ1 (Peprotech) and medium was collected, serially centrifuged at 300 *g* for 5 min, 2,000 *g* for 20 min, and 12,500 *g* for 20 min to remove cells, cell fragments and large EVs. Samples were then ultracentrifuged (UC) at 100,000 *g* for 70 min at 4°C, the EV-containing pellet was resuspended in PBS and UC again. The EV containing pellets were then resuspended in PBS and they were used for functional experiments or they were bound to latex beads (see section “Functional studies with EVs”).

### Nanoparticle Tracking Analysis (NTA)

Fibroblasts were cultured in FBS-free medium and the experiments were carried out in the same standard conditions (6-well plate, 100,000 cells/well, and 1.5 mL medium). Fibroblast supernatants were harvested after 2 days, 0.5 mL supernatants were serially centrifuged at 300 *g* for 5 min, 2,000 *g* for 20 min, and 12,500 *g* for 20 min. After centrifugation, 100 μL supernatant was diluted to 1 mL in PBS and particle size distribution and concentrations were measured on a ZetaView Z- Nanoparticle Tracking Analysis (NTA) instrument (Particle Metrix). For each measurement, 11 cell positions were scanned at 25°C. The following camera settings were used: auto expose, gain: 28.8, offset: 0, shutter: 100, and sensitivity: 80. The videos were analyzed with a minimum area of 5, maximum area of 1000 and a minimum brightness of 20 by the ZetaView Analyze software 8.05.10.

### Functional Studies With EVs

Ten μl (2.5 × 10^7^) EVs were added to each well with CRC organoids (20 μL Matrigel and 200 μL medium, 48-well tissue culture plates, Eppendorf). In some experiments, EVs were incubated with 10 μg/mL neutralizing anti-AREG antibody or control goat IgG (see Supplementary Information) for 1 h in 20 μL medium before applying them to organoids. When indicated, organoids were pre-incubated with the EGF receptor inhibitor gefitinib (5 μM, Tocris) 30 min before applying EVs and they were further cultured in the presence of the inhibitor.

### Semi-Quantitative Analysis of EVs by Anti-CD63 or Anti-CD81-Coated Beads

Conditioned media from fibroblasts, harvested after 2 days, were centrifuged at 300 *g* for 5 min and 2,000 *g* for 20 min. EVs were then bound to beads coated with anti-CD63 (Thermo Fisher, 10606D) or anti-CD81 (Thermo Fisher, 10616D). Before incubating with the samples, beads had been blocked with 0.1% BSA (Sigma) for 30 min at room temperature. 20 μL and 6 μL of the anti-CD63 or anti-CD81-coated beads were added to 200 μL supernatant, respectively. Beads were rotated overnight at 4°C, they were then magnetically separated, washed with PBS three times and they were labeled with FITC-anti-CD81, or PE-anti-CD63. The proportion of positive beads was detected by flow cytometry (FACSCalibur) and they were normalized to cell number (to 100,000 and 300,000 cells in case of CD63 and CD81-coated beads, respectively).

### Detection of AREG on EVs

EVs were separated from the FBS-free 2 days conditioned media of NCF or CAF cultures [5 × 10^5^ cells were grown in a 10 cm cell culture plate (Eppendorf)]. EV-containing pellets were resuspened in 500 μL PBS, they were incubated overnight at 4°C with 1 μL latex bead (Thermo Fisher # A37478, 3.9 g/100 mL), and blocked with 1% BSA (Sigma). Beads were then washed three times with PBS, they were divided into two parts and labeled with anti-CD63 PE or anti-AREG for 2 h at 4°C in 1% PBS. Anti-goat Alexa 488 was applied as secondary antibody for 1 h at 4°C. Samples were measured on a FACSCalibur (BD Biosciences, San Jose, CA, United States).

### Immunocytochemistry

Cells were fixed in 4% PFA for 20 min. They were then blocked and permeabilized in blocking buffer (PBS with 0.1% BSA, 5% FBS, and 0.1% Triton X–100). After washing with blocking buffer, cells were incubated with primary antibodies at 4°C overnight and then with secondary antibodies for 2 h at room temperature. The incubation steps were carried out in blocking buffer. After covering samples with ProLong Diamond antifade mountant countaining DAPI (Thermo Fisher, Waltham, MA, United States), they were analyzed with a Zeiss LSM800 confocal microscope.

### Whole-Mount Immunostaining

Colorectal cancer organoids were cultured in 8-well chamber slides (BD Biosciences, San Jose, CA, United States), fixed in 4% PFA for 30 min and washed with PBS. Blocking and permeabilization were carried out in whole-mount blocking buffer (WMBB, containing 5% FBS, 0.2% BSA, and 0.3% Triton X–100 in PBS) for 30 min. Samples were incubated with primary antibodies at 4°C overnight in WMBB. After washing in PBS + 0.3% Triton X–100 + 4% NaCl, labeled secondary antibodies were added overnight at 4°C. The organoids were then mounted with ProLong Diamond antifade mountant countaining DAPI (Thermo Fisher, Waltham, MA, United States) and analyzed with a Zeiss LSM800 confocal microscope. Images were evaluated by the ImageJ software. The antibodies used are listed in Supplementary Table S3.

### Transmission Electron Microscopy

The EV-containing pellet after UC and washing with PBS was resuspended in 20 μl PBS and a 2 μL droplet was dried on a 300 mesh grid (Electron Microscopy Sciences, United States). Fixing of EVs was carried out with 4% glutaraldehyde for 10 min and the grid was washed with water three times. EVs were treated with 2% phosphotungstic acid, they were dried at RT and images were captured with a MORGAGNI 268D (FEI, Netherlands) instrument.

### RNA Isolation and mRNA Measurements From Cells

RNA was isolated with the RNEasy Micro Kit (Qiagen, Hilden, Germany) according to the manufacturer’s protocol in 15 μL water. We measured RNA concentration with a NanoDrop instrument. Half μg RNA (in 20 μL final volume) was reverse transcribed with the SensiFAST^TM^ cDNA Synthesis Kit (Bioline, London, United Kingdom) and quantitative PCR reactions were carried out using the SYBRGreen method with the SensiFAST^TM^ SYBR^®^ Hi-ROX Kit (Bioline, London, United Kingdom). We used an ABI 7900HT Fast real-time PCR instrument in 384-well format in 5 μL volume. Results were evaluated with the following protocol: relative expression level = 2^–Δ*Ct*^, where ΔCt = Ct (gene of interest) – Ct (housekeeping gene). The primers are listed in Supplementary Table S4.

### TaqMan Low Density miRNA Array

Normal colon fibroblasts were cultured with our without 10 ng/ml TGFβ (6-well plate, 100,000 cells/well, and 1.5 mL medium). Fibroblast supernatants were harvested after 4 days, serially centrifuged at 300 *g* for 5 min, 2,000 *g* for 20 min, 12,500 *g* for 20 min, and the particle concentration was determined with NTA. Sample volumes with 5 × 10^8^ particles were supplemented with medium to 1 mL, EVs were isolated with anti-CD63 (40 μL), and anti-CD81 (12 μL)-coated beads O/N, they were washed 5 times with PBS and EVs were lyzed in Qiazol (Qiagen, Hilden, Germany). Total RNA with small RNAs was isolated with the miRNeasy Micro Kit (Qiagen, Hilden, Germany) according to the manufacturer’s instructions in 15 μL water. 2 μL total RNA was reverse transcribed with Megaplex RT primers, the samples were amplified with Megaplex PreAmp Primers and then TaqMan^TM^ Array Human MicroRNA A Cards v2.0 (all from Thermo Fisher, Waltham, MA, United States) were used according to the manufacturer’s protocol. Array cards were measured with an ABI 7900HT instrument and Ct < 40 was regarded as miRNA present in the sample. We then selected miRNAs that were absent in all the three controls and present in the TGFβ-treated samples (*n* = 3). As background measurements, samples isolated from cell-free cultures were applied (*n* = 2).

### Statistical Analysis

Kruskal–Wallis with Dunn *post hoc* test, Mann–Whitney *U*-test or Student’s paired or unpaired *t*-tests were used with ^∗^*p* < 0.05, ^∗∗^*p* < 0.01, and ^∗∗∗^*p* < 0.005 significance levels. Microsoft Excel, SPSS version 25 and GraphPad softwares were used for statistical evaluation. Mean + SD or median and 25 percentile values are shown with *n* = 3–5 biological replicates, unless otherwise indicated.

## Results

### Fibroblast Activation Does Not Modify EV Release Intensity

**FIGURE 1:**
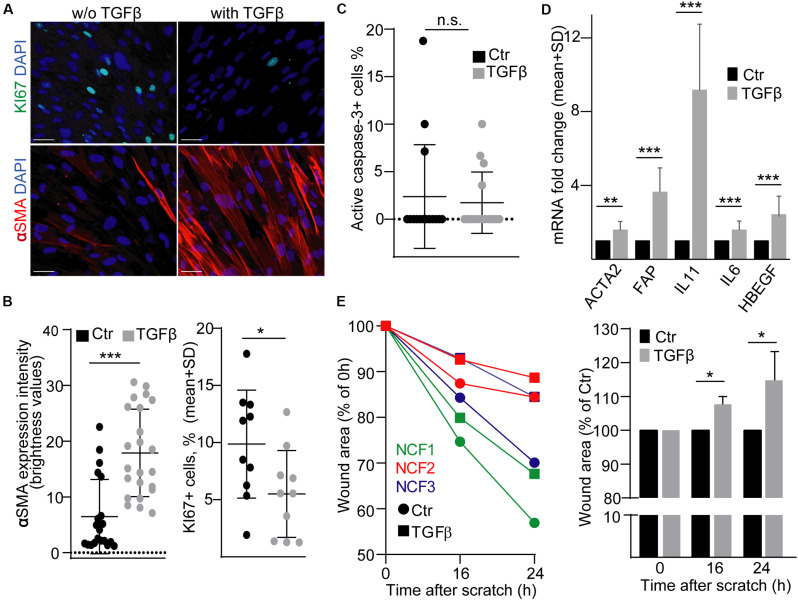
TGFβ activates normal colon fibroblasts (NCF). **(A,B)** KI67 and αSMA immunostaining of NCFs with or without TGFβ (4 days). Confocal microscopic images **(A)** and their quantification **(B)**. αSMA intensity was determined by ImageJ software. **(C)** The proportion of active caspase-3+ apoptotic control or TGFβ-treated NCFs, based on immuncytochemistry, and confocal microscopic images. **(D)** mRNA fold change of the indicated genes in NCFs (RT-qPCR, *n* = 6, paired samples). **(E)** Relative wound area compared to the initial area (right) and to the area of the untreated samples at each time point (left) in confluent fibroblast cultures (*n* = 3, paired samples). Data were obtained from three repeated experiments in **(B,C)**. Mann–Whitney *U*-test **(B,C)** or paired *t*-test **(D,E)** were used with *p < 0.05, **p < 0.01, and ***p < 0.005. Scale bars: 50 μm **(A)**.

Fibroblasts in tumors show a characteristic gene expression profile and thus, these CAFs are considered to be in an activated state. TGFβ is critically involved in colon fibroblast activation, e.g., it induces the initial steps of metastasis formation by inducing IL-11 secretion from fibroblasts (Calon et al., 2012). To study the effects of EVs released by activated fibroblasts on CRC cells, first we used a widely accepted and reproducible, commercially available model system, normal human colon-derived fibroblasts (NCF), and we activated them by TGFβ. As previously published, TGFβ reduced the proportion of the KI67+ proliferating cells (Calon et al., 2015) and increased the intensity of αSMA expression, a general marker of intestinal myofibroblasts (Figures 1A,B). Importantly, we found no change in the percentage of active caspase-3+ apoptotic cells (Figure 1C). As expected, we observed an increased RNA level of *FAP*, *ACTA2* (encoding αSMA), *IL6*, *IL-11*, and *HBEGF*, thus, proving that TGFβ treatment led to characteristic transcriptional changes (Figure 1D). Importantly, TGFβ resulted in larger wound area in a wound healing assay (Figure 1E), showing the reduced migration of NCFs.

**FIGURE 2:**
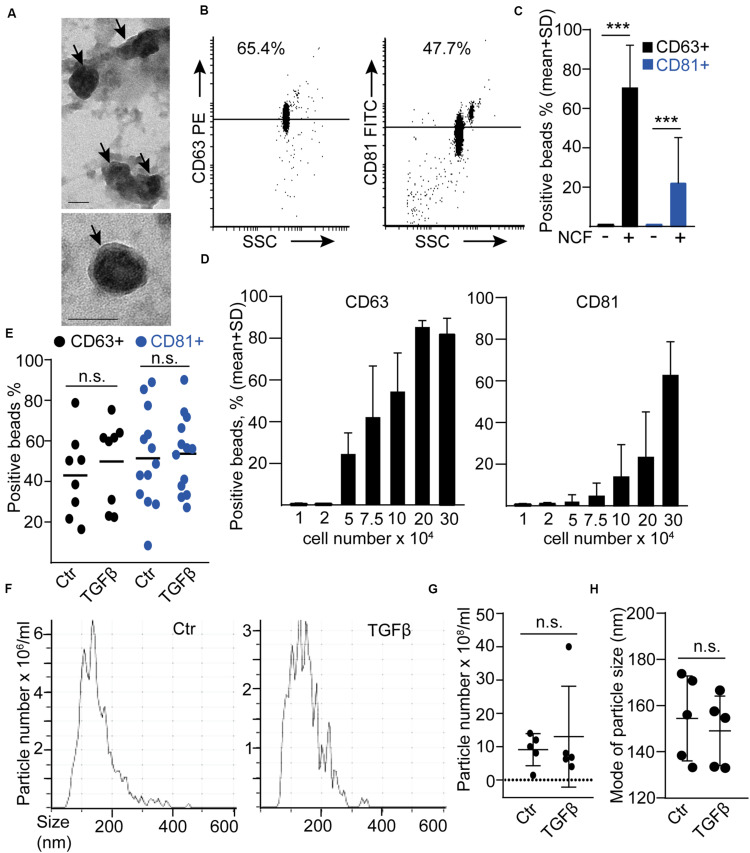
TGFβ has no effect on EV release from normal colon fibroblasts (NCF). **(A)** Transmission electronmicroscopic (TEM) images of the UC pellet derived from NCF supernatant. The arrows show EVs. **(B,C)** Detecting EVs from NCF supernatants with anti-CD81 or anti-CD63-coated beads. Flow cytometric images **(B)** and quantification of the data **(C)**. Serum-free medium (NCF-) samples were compared to NCF supernatants (NCF+; *n* = 3). **(D)** The percentage of anti-CD63 or anti-CD81-coated positive beads after incubation in supernatants from NCF cultures with different cell numbers (flow cytometry, *n* = 4). **(E)** The percentage of positive beads after incubating them in control or TGFβ-treated (10 ng/ml, 4 days) NCF supernatant (normalized to cell number). **(F–H)** Representative images **(F)**, the concentration of particles (**G**, normalized to 100,000 cells), and their mode size **(H)** in control and TGFβ-treated NCF supernatants (NTA). Mann–Whitney *U*-test **(E,G,H)** or paired *t*-test **(C)** were used. Scale bars: 50 nm **(A)**. ***p < 0.005.

Similarly to our previous results (Oszvald et al., 2020), EVs were detected in NCF conditioned medium by transmission electron microscopy (TEM; Figure 2A). In addition, we also detected EVs in these samples when using anti-CD63 or anti-CD81-coated beads and flow cytometry (Figures 2B,C). CD81 and CD63 are markers characteristic for EVs, thus, this bead-based method collects EVs semi-quantitatively from cell culture supernatants (Figure 2D; Ostrowski et al., 2010; Szvicsek et al., 2019). By using this method, we did not find an increased CD81+ or CD63 + EV release after TGFβ (Figure 2E). Similarly, NTA, a widely used method for quantifying EV amounts, showed no difference in particle concentration and no shift in particle size distribution after treatment (Figures 2F–H). Importantly, all these data have been normalized to cell number, excluding the possibility that changes in the percentage of the proliferating cells after TGFβ treatment could influence our conclusions. Thus, these results indicate that TGFβ-induced NCF activation did not have a major effect on EV secretion.

### TGFβ-Induced Fibroblast Activation Modifies the miRNA Cargo of EVs

**FIGURE 3:**
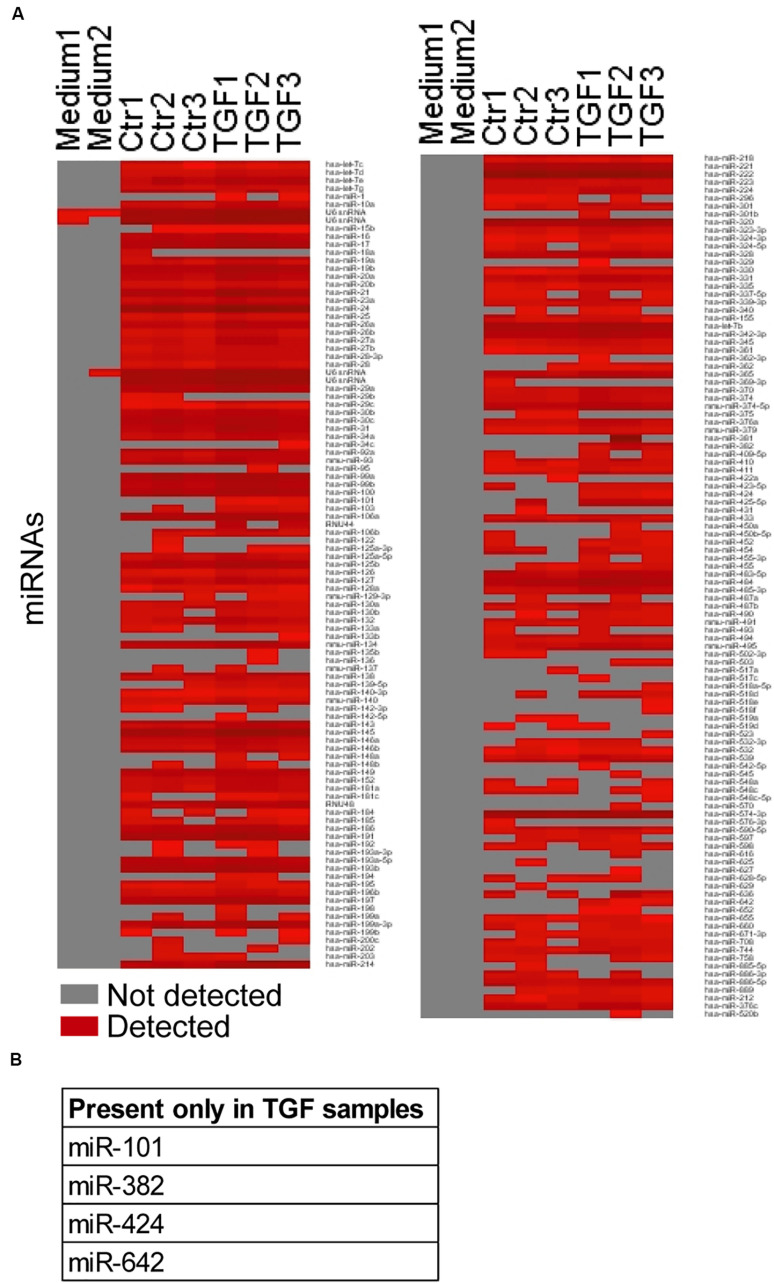
TGFβ modifies the miRNA profile of NCF-derived fibroblasts. **(A)** The presence/absence of miRNAs in the untreated/treated NCF EV samples. Note that only miRNAs that were present in at least one of the samples are shown. **(B)** miRNAs present in all TGFβ-treated samples and absent in all controls.

To determine changes in the EV cargo after NCF activation, we first focused on miRNAs that have a known important role in EV-mediated intercellular communication. Previously we proved that miRNAs from EVs isolated by antibody-coated beads had less unspecific miRNA background compared to other methods. Thus, we applied anti-CD63-coated and anti-CD81-coated beads for our miRNA analysis (Szvicsek et al., 2019). The medium-scale screen, analyzing 377 miRNAs, detected 209 miRNAs in at least one of the six samples measured (Figure 3A and Supplementary Table S5). Interestingly, we found that four miRNAs (hsa-miR-101, 382, 424, and 642) were present only in TGFβ-treated EV samples (Figure 3B and Supplementary Table S5). Thus, NCF activation changes the miRNA profile of the EV cargo.

### Fibroblast-Derived EVs Induce CRC Cell Proliferation via Transporting AREG Independently of TGFβ Activation

**FIGURE 4:**
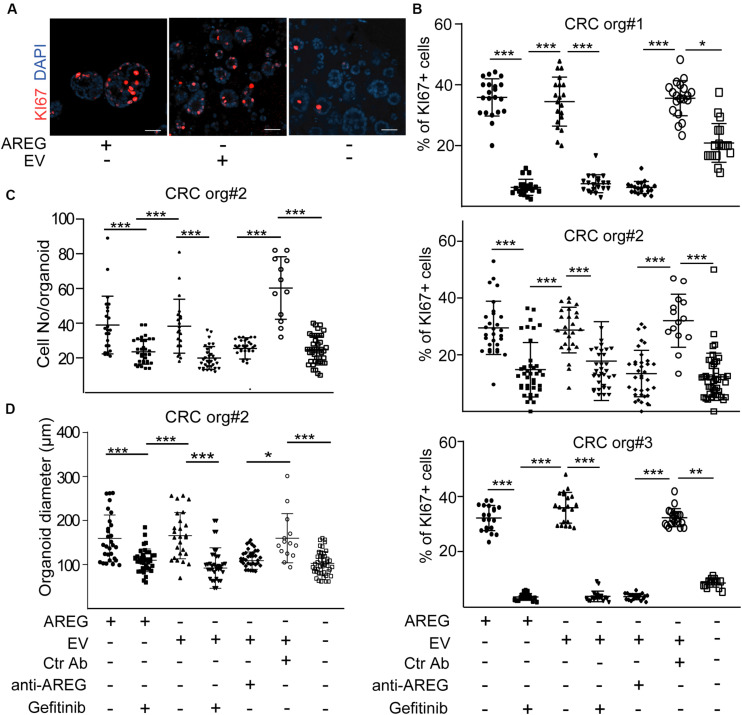
NCF-derived EVs induce the proliferation of CRC organoid cells via carrying AREG. **(A,B)** Representative confocal microscopic images **(A)** and the quantification of KI67+ cells in CRC patient-derived organoid lines with the indicated treatments. Note that 2.5 × 10^7^ NCF-derived EVs were applied. **(C,D)** Cell number **(C)** and diameter **(D)** of CRC organoids Kruskal–Wallis and Dunn’s multiple comparison tests were used **(B,C,D)** with *p < 0.05, **p < 0.01, and ***p < 0.005. Scale bars: 100 μm **(A)**. Confocal microscopic images were quantified from three experiments for each organoid line for **(B–D)**.

**FIGURE 5:**
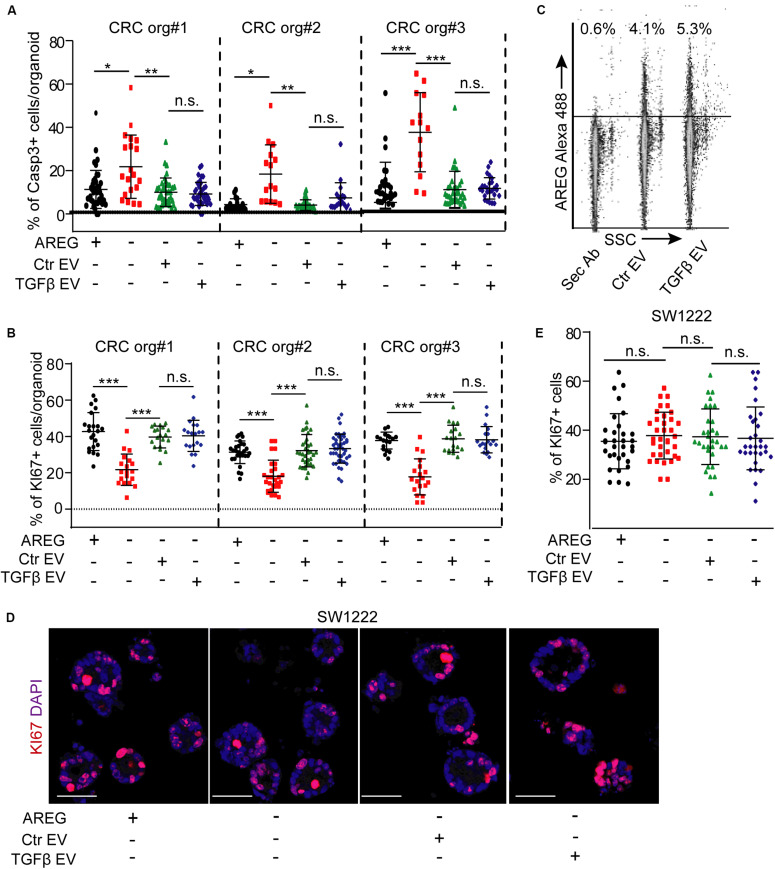
TGFβ does not modify the effects of EVs either in EGF-dependent or in EGF-independent 3D models. **(A)** The percentage of active caspase-3+ cells in CRC patient-derived organoid lines in the presence or absence of AREG, control, or TGFβ-treated NCF-derived EVs. **(B)** The effect of NCF-derived EVs on CRC organoids. NCFs were pre-treated with TGFβ (TGFβ EV) or they were cultured untreated (Ctr EV) before collecting EVs. **(C)** Detecting AREG on EVs isolated from control (Ctr EV) or TGFβ-treated NCFs (TGFβ EV) and bound to latex beads (flow cytometry). **(D,E)** The percentage of KI67+ cells in SW1222 cell-derived colonies in the presence of the indicated treatments. Representative confocal microscopic images **(D)** and their quantification **(E)**. EVs were isolated from control or TGFβ-treated (4 days) NCF cultures. Kruskal–Wallis and Dunn’s multiple comparison tests were used **(A,B,E)**. Confocal microscopic images were quantified from three experiments for **(A,B,E)**. Scale bars: 50 μm **(D)**. *p < 0.05, **p < 0.01, ***p < 0.005.

Normoxic fibroblast-derived EVs had no effect on the colony formation of CRC organoid cells in the presence of EGF (Szvicsek et al., 2019). In addition, we proved earlier that at least one of the EGF family members, AREG, travels via fibroblast-derived EVs (Oszvald et al., 2020). Thus, EVs may be important for EGF-dependent organoids with wild type KRAS and BRAF in the absence of external EGF family members, such as AREG. We first added NCF-derived EVs to patient-derived organoid lines known to be dependent on EGF [(Szvicsek et al., 2019) and see section “Human colon organoid cultures”]. Importantly, we observed a marked reduction in the percentage of KI67+ proliferating organoid cells in the absence of AREG which was restored by NCF-derived EVs (Figures 4A,B). In addition, blocking EGF receptor by the EGF receptor inhibitor gefitinib or pre-incubating EVs with a neutralizing anti-AREG antibody inhibited the effects of EVs (Figures 4B–D), thus, proving the critical role of EV-bound AREG on CRC cell proliferation. In addition, the proportion of active caspase-3+ apoptotic organoid cells increased in the absence of AREG and this was restored by control or TGFβ-treated NCF-derived EVs (Figure 5A). Interestingly, we observed no difference in the percentage of KI67 + cells when CRC organoids were treated with identical numbers of EVs isolated from control or TGFβ-activated NCFs (Figure 5B). Importantly, AREG was present on both control and treated NCF-derived EVs, detected by latex beads that bind EVs (Figure 5C). Thus, although TGFβ resulted in a change of EV miRNA cargo, this had no effect on CRC cell proliferation and both control and activated fibroblast-derived EVs induced CRC proliferation via AREG in organoids that are dependent on EGF family members.

### EVs do Not Critically Modify the Proliferation of EGF-Independent CRC Cells

We next tested a 3D model system of CRC cells that are independent of EGF activity. The SW1222 cells form 3D organoid-like structures and the larger megacolonies contain lumens (Yeung et al., 2010, 2011). Importantly, the presence and the number of lumens correlate with CRC stem cell activity and differentiation. Furthermore, megacolonies highly resemble organoids (Yeung et al., 2010), and these cells do not require EGF family members. As expected, recombinant AREG had only a marginal inducing effect on the proportion of KI67 + cells and neither control nor TGFβ-activated NCF-derived EVs modified the ratio of proliferating cells (Figures 5D,E). These data suggest that EVs released by fibroblasts have no effect on CRC proliferation when EGF activity is dispensible.

### TGFβ Does Not Modify EV Release in CRC Patient-Derived Fibroblasts

**FIGURE 6:**
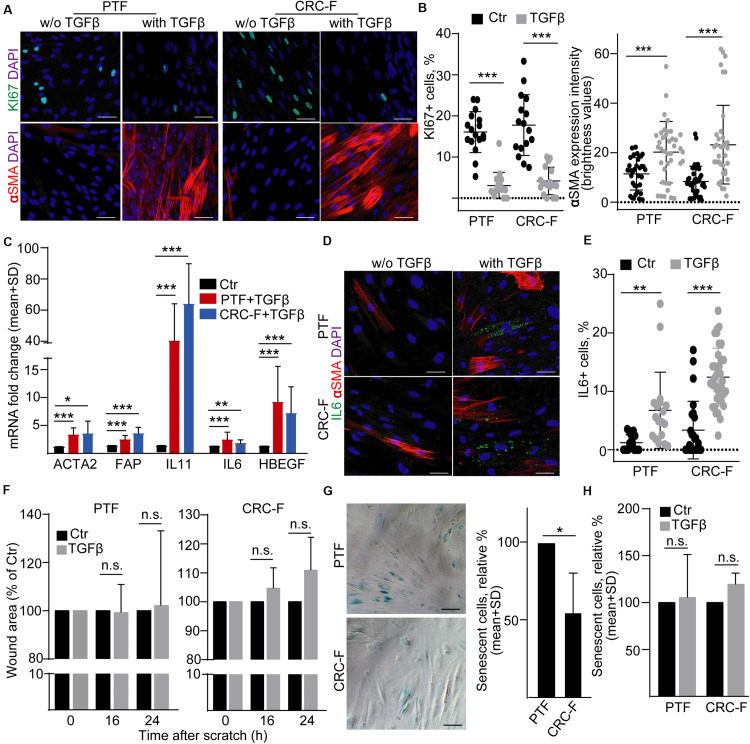
TGFβ activates PTFs and CRC-Fs. **(A,B)** αSMA and KI67 immunostaining in control and TGFβ-treated (4 days) PTFs or CRC-Fs (confocal microscopy; **A**) and their quantification **(B)**. αSMA expression intensity was determined by the ImageJ software. **(C)** Fold change of the indicated mRNAs in TGFβ-treated fibroblasts compared to untreated controls (RT-qPCR, *n* = 4). Note that pairwise comparisons were carried out between control and treated samples. **(D)** IL-6 and αSMA detection in PTFs and CRC-Fs (confocal microscopy). **(E)** The proportion of IL-6+ cells with the indicated treatments (quantification of confocal microscopy images). **(F)** Relative wound area of the TGFβ-treated fibroblasts compared to the control confluent fibroblast cultures (*n* = 3). **(G–H)** The proportion of cells with SA-β-galactosidase activity. Representative microscopic images **(G)** and their quantification (**G-H**, *n* = 3, paired samples). Mann–Whitney *U*-test **(B,E)** or paired *t*-test **(C,F,G,H)** were used with *p < 0.05, **p < 0.01, and ***p < 0.005. Scale bars: 50 μm **(D,G)**.

**FIGURE 7:**
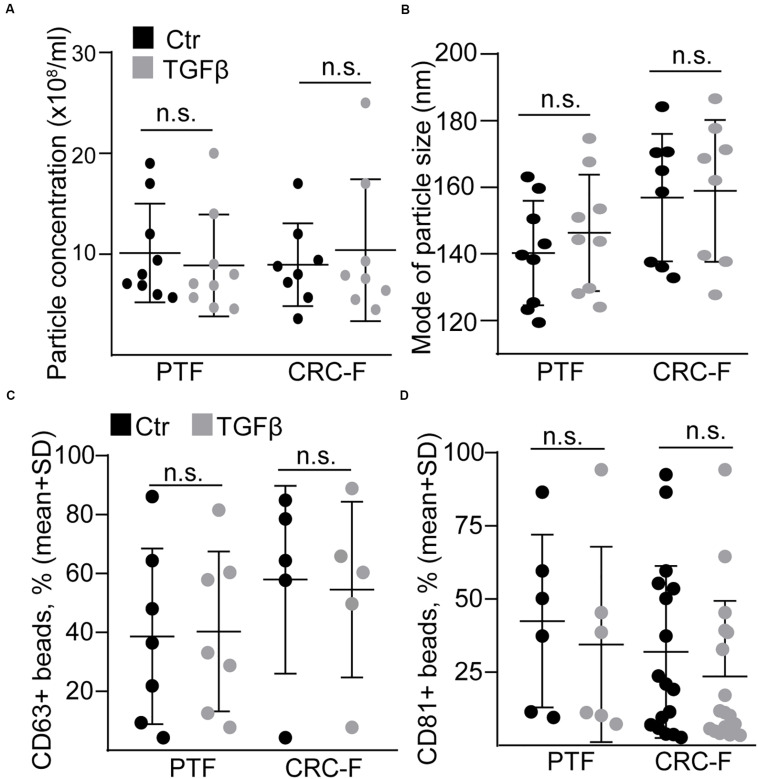
TGFβ does not modify EV release from peritumoral (PTF) or tumor (CRC-F) fibroblasts. **(A,B)** The concentration (**A**, normalized to 100,000 cells) and mode size of the particles **(B)** from PTF or CRC-F cells with or without pre-treatment with TGFβ (10 ng/ml, 4 days), measured by NTA. **C,D**) The percentage of anti-CD63 **(C)** or anti-CD81-coated **(D)** positive beads (normalized to cell number, see section “Semi-quantitative analysis of EVs by anti-CD63 or anti-CD81-coated beads”) that were incubated in supernatants from the indicated cells. Mann–Whitney *U*-test was used.

To prove our findings in another model system, we established cultures from the peritumoral (normal) colon segment (PTF) and the CRC tissue (CRC-F) of patients. Similarly to NCFs, TGFβ reduced the proportion of the proliferating cells and increased the intensity of αSMA expression, a general marker of intestinal myofibroblasts, in both CRC-Fs and PTFs (Figures 6A,B). Furthermore, we observed an increased RNA level of *FAP*, *ACTA2*, *IL-6*, *IL-11*, and *HBEGF* (Figure 6C). Unlike NCFs, TGFβ induced the appearance of IL-6 + cells both in PTF and CRC-F cultures (Figures 6D,E), resulting in the heterogeneity in fibroblast cultures. To further characterize the response of the fibroblast cultures to TGFβ, we carried out wound healing assay. Interestingly, we observed no difference in the wound area after TGFβ treatment in either PTFs or CRC-F cultures (Figure 6F). In addition, CRC-Fs showed a lower percentage of cells positive for SA-β-galactosidase activity, a widely used marker for senescence, compared to their paired PTFs (Figure 6G). TGFβ had no effect on the precentage of senescent cells in either fibroblast subpopulations (Figure 6H). Importantly, TGFβ-induced fibroblast activation did not modify the amount of secreted EVs either in PTFs or in CRC-Fs, as detected by NTA, with anti-CD63 or anti-CD81-coated beads (Figures 7A–D). Thus, we found that TGFβ induced similar changes both in PTFs and in CRC-Fs.

### CRC-F-Derived EVs Induce CRC Proliferation in EGF-Dependent Patient-Derived Organoid Lines

**FIGURE 8:**
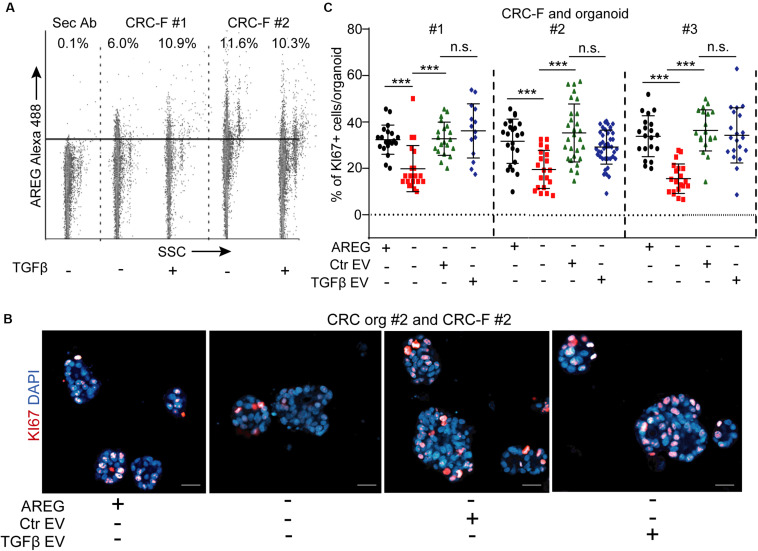
CRC-F cell-derived EVs increase the percentage of proliferating CRC organoid cells independently of pre-treatment with TGFβ. **(A)** Detecting AREG on EVs isolated from control or TGFβ-treated CRC-F cells and bound to latex beads (flow cytometry). **(B,C)** The percentage of KI67+ CRC organoid cells with or without AREG or EVs derived from control or treated CRC-Fs (paired fibroblast and organoid samples). Confocal microscopic images **(B)** and quantification **(C)**. Kruskal–Wallis and Dunn’s multiple comparison tests **(C)** were used. Scale bars: 50 μm. ***p < 0.005.

Since we found no difference between our PTF and CRC-F cultures in their activation, we focused on CRC-F fibroblasts. Similarly to NCFs, we detected AREG on both control and TGFβ-treated CRC-F-derived EVs by flow cytometry (Figure 8A) and these EVs restored the proportion of KI67 + CRC cells in EGF-dependent organoids in the absence of exogenously added EGF family members (Figures 8B,C). Importantly, we observed no difference when CRC-F cultures had been pre-treated with TGFβ and identical numbers of EVs were applied (Figures 8B,C). Thus, our results show that fibroblast-derived EVs induce CRC proliferation via an activation-independent mechanism only in EGF-dependent CRC organoids.

## Discussion

In this study we prove that the TGFβ-induced activation of NCFs modifies EV miRNA cargo and hsa-miR-101, 382, 424, and hsa-miR-642 are present only in EVs derived from activated NCFs. In addition, TGFβ results in gene expression changes in CRC-Fs as well. However, fibroblast-derived EVs induced CRC cell proliferation of EGF-dependent patient-derived organoids via carrying amphiregulin AREG, independently of the TGFβ-induced EV cargo change and fibroblast activation. In addition, EVs had no role in a 3D system where EGF activity was not required.

Although stromal cells, such as fibroblasts, are critical in the progression of many tumor types, their exact activation mechanism and the role of fibroblast subpopulations is still poorly known. As an example, pancreatic ductal adenocarcinoma (PDAC), an extremely aggressive cancer with an accumulation of stromal elements, contains myCAF and iCAF fibroblast cells (Ohlund et al., 2017; Biffi et al., 2019). Whereas fibroblasts with contact to tumor cells express αSMA and differentiate to myCAFs, peripheral cells produce IL–6 as iCAFs (Ohlund et al., 2017). In addition, TGFβ inhibits the differentiation to iCAFs, thus, this cytokine plays a central role in shaping the activation pattern of CAFs (Biffi et al., 2019). Interestingly, a previous publication found that IL-6 + fibroblasts are present in CRC as well (Huynh et al., 2016). In line with these results, we provide evidence that TGFβ is critically involved in establishing this fibroblast heterogeneity both in PTFs and in CAFs. The importance of cellular heterogeneity has also been proven in normal human colon fibroblasts by identifying subpopulations with differential expression profile (Kinchen et al., 2018). Interestingly, TGFβ did not result in the appearance of IL-6 + fibroblasts in NCFs in our experiments, however, other profile changes may have occured that we have not tested in our work.

Peri-tumoral fibroblasts are often used as normal fibroblast controls (Berdiel-Acer et al., 2014). However, a recent study found only a limited amount of difference in gene expression between PTFs and CRC-Fs and αSMA, generally considered as a marker of activated fibroblasts, was present in PTFs as well (Berdiel-Acer et al., 2014). Furthermore, TGFβ is considered as a major inducer of fibroblast activation in CRC (Calon et al., 2012), leading to the expression of characteristic genes, such as *IL-11* (Calon et al., 2012). Interestingly, both PTFs and CRC-Fs produced more *IL-11*, *ACTA2, HBEGF, IL-6*, and *FAP* when they were treated with TGFβ, suggesting that these cells can be further activated *in vitro*. In our experiments, PTFs and CRC-Fs behaved similarly in the tested parameters after TGFβ treatment, such as cellular heterogeneity, gene expression changes, wound size in wound healing assays or cell senescence. Thus, these results show that either PTFs were already in a pre-activated state or CRC-Fs lost their activated features in our system.

Not only CRC cell-derived EVs, but also EVs released by stromal cells may be important in tumorigenesis. Inhibiting ELFN1-AS1 long non-coding RNA in umbilical cord mesenchymal stem cell-derived EVs suppresses colon adenocarcinoma proliferation and migration in cell lines (Dong et al., 2019). Furthermore, granulocytic myeloid-derived suppressor cells release EVs that promote the stem cell-like properties of CRC cells through S100A9 (Wang et al., 2019). In addition, EVs secreted by macrophages polarized to the M2 direction induce CRC cell migration (Lan et al., 2019). Interestingly, in two elegant studies fibroblast-derived EVs have been proven to promote resistance for chemotherapeutic drugs in CRC (Hu et al., 2015; Hu J. L. et al., 2019) and by using cell lines and xenografts, the authors found that the effect was accomplished by dedifferentiation of CRC cells to cancer stem cells via Wnt proteins (Hu Y. B. et al., 2019). Previously we found that normoxic fibroblast-derived EVs did not affect the colony number of CRC organoids in the presence of EGF (Szvicsek et al., 2019). In our present study we identified a novel mechanism how fibroblast-derived EVs induce CRC tumorigenesis by carrying AREG when EGF is absent from the culture medium. So far all previously published works used cell lines and xenografts. Importantly, we used patient-derived organoids in our studies that is one of the best current models for human cancers by maintaining the intra-patient tumor cell heterogeneity. Since we selected CRC organoids for mutations in the Wnt pathway by removing the Wnt agonist R-Spondin1 from organoid culture medium, our surviving organoid lines did not require external Wnt proteins, thus, the effect of EV-bound Wnt proteins could not be studied in our system. CAF-derived EVs promote metastasis and epithelial-mesenchymal transition (EMT) by miR-92a-3p and this effect was more pronounced compared to control fibroblasts (Hu J. L. et al., 2019). Interestingly, we found a change in the EV cargo with miR-101, 382, 424, and 642 present only in EVs released by TGFβ-treated activated fibroblasts. The role of these miRNAs in CRC proliferation and apoptosis is controversial. Whereas miR-101 has an anti-proliferative effect (Sastre et al., 2019; Shao et al., 2019) the level of miR-382 is elevated in CRC and it induces tumor progression (Savardashtaki et al., 2019). Importantly, organoids are routinely cultured in the laminin-rich 3D matrix Matrigel which is not suitable to model invasion and metastasis. Thus, AREG transported by EVs is critical in inducing cell proliferation in organoids that are dependent on EGF activity, however, changes in the miRNA EV cargo after fibroblast activation is not central in inducing CRC proliferation and it may become important in other steps of CRC tumorigenesis. Furthermore, in contrast to the effect of miR-92a-3p (Hu J. L. et al., 2019), we found no difference in CRC cell proliferation or apoptosis when using normal fibroblasts, CRC-Fs or when these cells were pre-treated with TGFβ, suggesting that the AREG-mediated EV effect is independent of fibroblast activation.

The EGF ligand family members are synthesized as membrane-bound molecules and they are cleaved by proteases to result in soluble molecules. However, many EGF family members, such as AREG may act in its membrane-bound uncleaved form in a juxtacrine manner as well (Singh and Harris, 2005; Berasain and Avila, 2014). Indeed, several studies have identified unprocessed AREG on EVs and proved its EV-bound activity (Higginbotham et al., 2011; Taverna et al., 2017; Raimondo et al., 2019; Zhang et al., 2019). We previously published that AREG transported by fibroblast-derived EVs plays an important role in shaping the intestinal stem cell niche by providing EGF activity (Oszvald et al., 2020). Importantly, EVs can be taken up into the target cells with endocytosis or macropinocytosis and this process is induced by EGF receptor activation (Nakase et al., 2015). In addition, activated EGF receptor is internalized with the EGF ligand and they continue stimulating signaling pathways even after endocytosis (Tomas et al., 2014). Thus, this raises the possibility that EVs induce CRC cell proliferation via cell surface binding between AREG and EGF receptor, and/or after endocytosis. Although further studies will be needed to decide the exact mechanism, we provide here evidence that EV-bound AREG critically induces CRC cell proliferation when CRC cells have an unmutated EGF signaling pathway.

Collectively, by using the 3D organoid model, we show that fibroblast-derived EVs transport AREG and they increase the number of proliferating CRC cells in patient-derived organoid lines that are dependent on external EGF activity, but not in an EGF-independent model system. Although NCFs activated by TGFβ secrete EVs with a characteristic difference in their miRNA content compared to controls, we found no difference in the effect of EVs when NCFs were pre-activated by TGFβ. Similarly, EVs from CRC-Fs pre-activated by TGFβ did not modify the proportion of proliferating CRC organoid cells. All these data point to the critical and activation-independent role of AREG transported by fibroblast-derived EVs at early stages of CRC tumorigenesis when the EGF signaling pathway is not yet mutated.

## Data Availability Statement

All datasets presented in this study are included in the article/Supplementary Material.

## Ethics Statement

The studies involving human participants were reviewed and approved by Ethics Committee of the Medical Research Council of Hungary (ETT-TUKEB, https://ett.aeek.hu/en/secretariat/, 7-8 Széchenyi István tér, Budapest, H-1051, Hungary). The patients/participants provided their written informed consent to participate in this study.

## Author Contributions

ÁO and ZS: Conception and design, collection, and assembly of data, data analysis and interpretation, and manuscript writing. AB, KD, and TT: Provision of study material. MP, AK, and ZV: Data collection. EB: Data interpretation and writing the manuscript. ZW: Conception and design, financial support, data analysis and interpretation, manuscript writing, and final approval of the manuscript. All authors contributed to the article and approved the submitted version.

## Conflict of Interest

The authors declare that the research was conducted in the absence of any commercial or financial relationships that could be construed as a potential conflict of interest.
