# Supplementary material for: Fibroblast-Derived Extracellular Vesicles Induce Colorectal Cancer Progression by Transmitting Amphiregulin
Source: Front Cell Dev Biol. 2020 Jul 7;8:558. doi: 10.3389/fcell.2020.00558 (PMC7381355; doi:10.3389/fcell.2020.00558)
Supplement: Supplementary file 1 [file Data_Sheet_1.pdf]

Fibroblast-derived extracellular vesicles induce colorectal cancer progression by transmitting amphiregulin

Patient	PTF	CRC-F	Org	Age	Gender	Diagnosis	TNM	Grade
1	-	+	+	63	F	adenocarcinoma	T3N0M0	2
2	+	+	+	74	M	adenocarcinoma	T3N0M0	2
3	-	+	+	75	M	adenocarcinoma	T3N2aM0	2
4	+	+	-	52	M	adenocarcinoma	T3N0M1	2
5	+	+	-	73	F	adenocarcinoma	T3N0M0	2
6	+	+	-	77	F	adenocarcinoma	T2N1aM0	2
7	+	+	-	76	M	adenocarcinoma	T3N1bM0	2
8	+	+	-	73	M	adenocarcinoma	T3N0M0	2
9	-	+	-	45	F	adenocarcinoma	T3N2bM0	2
10	-	+	-	69	F	adenocarcinoma	T2N0M0	1
11	+	-	-	42	M	adenocarcinoma	T4N2aM0	2
12	+	-	-	69	M	adenocarcinoma	T4N0M0	1
13	+	-	-	49	M	adenocarcinoma	T3N2aM0	1

Table S1. Patient data for PTF and CRC-F cultures. Note that CRC organoids #1-3 have been published in our previous studies (Szvicsek et al., 2019).

Figure No	PTF samples	CRC-F samples	Comment
Fig 6B	4, 5, 6, 7, 11	4, 5, 6, 7, 10	3-4 images/sample evaluated
Fig 6C	6, 7, 11, 12	6, 7, 9, 10	
Fig 6E	4, 5, 6, 7, 11	4, 5, 6, 7, 10	4-5 images/sample evaluated
Fig 6F	4, 6, 8	4, 6, 8	
Fig 6G, H	4, 5, 6	4, 5, 6	
Fig 7A, B	2, 4, 5, 6, 7, 8, 11, 12, 13	2, 4, 5, 6, 7, 8, 9, 10	
Fig 7C	4, 5, 6, 7, 8, 11, 12	4, 5, 6, 7, 8	
Fig 7D	4, 5, 6, 7, 8, 11	4, 5, 6, 7, 8, 9	For CRC-F, measurements were repeated three times
Fig 8C	N/A	1, 2, 3	Each dot represents one organoid

Table S2. Samples used in different experiments.

Antibody	Source	Clone/Cat No
FITC anti-human CD81	Molecular Probes	A15753
PE anti-human CD63	Sigma	SAB4700218
anti-KI67	Abcam	Ab16667
anti-human/mouse active caspase-3	R&D Systems	AF835

anti-human αSma	Sigma	A5228
anti-human AREG	R&D Systems	AF262
anti-human CD81	Sigma	SAB3500454
control goat IgG	R&D Systems	AB108
anti-mouse IgG Alexa 488	Invitrogen/Thermo Fisher	AF21202
anti-mouse IgG Alexa 568	Invitrogen/Thermo Fisher	AF10037
anti-rabbit IgG Alexa 488	Invitrogen/Thermo Fisher	A21206
anti-rabbit IgG Alexa 568	Invitrogen/Thermo Fisher	A11011
anti-goat IgG Alexa 488	Invitrogen/Thermo Fisher	A21467
anti-goat IgG Alexa 568	Invitrogen/Thermo Fisher	A11057S

Table S3. Antibodies used in our experiments.

Primer name	Sequence	Sequence ID
hACTA2_fw	CTGACCCTGAAGTACCCGAT	ENSG00000107796
hACTA2_rev	GTCATTTTCTCCCGGTTGGC	
hFAP_fw	GGAAATGAGCTTCCTCGTCC	ENSG00000078098
hFAP_rev	GGTGGATCTCCTGGTCTTTG	
hIL11_fw	GACAAATTCCCAGCTGACGG	ENSG00000095752
hIL11_rev	CGCAGGTAGGACAGTAGGT	
hIL6_fw	ATTCCAAAGATGTAGCCGCC	ENSG00000136244
hIL6_rev	AGTGCCTCTTTGCTGCTTTC	
hHBEGF_fw	TTATCCTCCAAGCCACAAGC	ENSG00000113070
hHBEGF_rev	CCCATGACACCTCTCTCCAT	
hHPRT1_fw	TGAGGATTTGGAAAGGGTGT	ENSG00000165704
hHPRT1_rev	TCCCCTGTTGACTGGTCATT	

Table S4. The sequence of primers used for RT-qPCR.

Table S5. Ct values detected by TaqMan miRNA array cards from EVs isolated by anti-CD63 and anti-CD81-coated beads. Medium control, untreated and TGFβ-treated NCF samples were applied (separate Excel sheet).

Supplementary References

Szvicsek, Z., Oszvald, A., Szabo, L., Sandor, G.O., Kelemen, A., Soos, A.A., et al. (2019). Extracellular vesicle release from intestinal organoids is modulated by Apc mutation and other colorectal cancer progression factors. Cell Mol Life Sci. 76, 2463-2476.
